# Supplementary material for: Performance Characteristics of a Novel 3D-Printed Bubble Intermittent Mandatory Ventilator (B-IMV) for Adult Pulmonary Support
Source: Bioengineering (Basel). 2022 Apr 2;9(4):151. doi: 10.3390/bioengineering9040151 (PMC9027535; doi:10.3390/bioengineering9040151)
Supplement: Supplementary file 1 [file bioengineering-09-00151-s001.zip › bioengineering-1651297-supplementary.pdf]

bioengineering

MDPI

Performance Characteristics of a Novel 3D-Printed Bubble Intermittent Mandatory Ventilator (B-IMV) for Adult Pulmonary Support [Supplement]

Jonathan A. Poli [1,*], Christopher Howard [2,*], Alfredo J. Garcia III [3], Don Remboski [3], Peter Littlewood [3], John P. Kress [3], Narayanan Kathsuri [3], Alia Comai [3], Kiran Soni [3], Philip Kennedy [3], John Ogger [3] and Robert M. DiBlasi [1,4]

Contents

1. Introduction to Supplement 1

2. Historical Development of BubbleVent 2

3. ISO 80601 Pressure Ventilation Testing 10

1. Introduction to Supplement

The COVID-19 pandemic, caused by the novel coronavirus (SARS-CoV-2), has led to over five million deaths worldwide at the time of writing, with the global weekly death toll remaining in the thousands. In the worst cases, COVID-19 patients exhibit a form of severe acute respiratory distress syndrome (ARDS) that requires mechanical ventilation to stabilize gas exchange while minimizing the respiratory effort.

Early on during the COVID-19 pandemic, it was projected that the need for mechanical ventilation would vastly exceed the number of mechanical ventilator devices available during hospital surge capacity. This anticipated need led to substantial and widespread efforts to develop and manufacture devices for emergency respiratory support. The BubbleVent device was conceived as a simple, low-cost, disposable critical care ventilator to address this potential acute ventilator shortage. The BubbleVent had no pre-existing design; thus, it was novel work undertaken by a small team from industry, medical practice, and academia over the course of approximately 6 months.

The purpose of this Supplement is to provide the reader with a deeper understanding of the design process behind the BubbleVent. Each iteration of the design was a new step towards the final prototype. At the end, benchmark testing of the BubbleVent against the ISO 80601 standard with a critical care ventilator was performed to demonstrate its performance.

2. Historical Development of BubbleVent

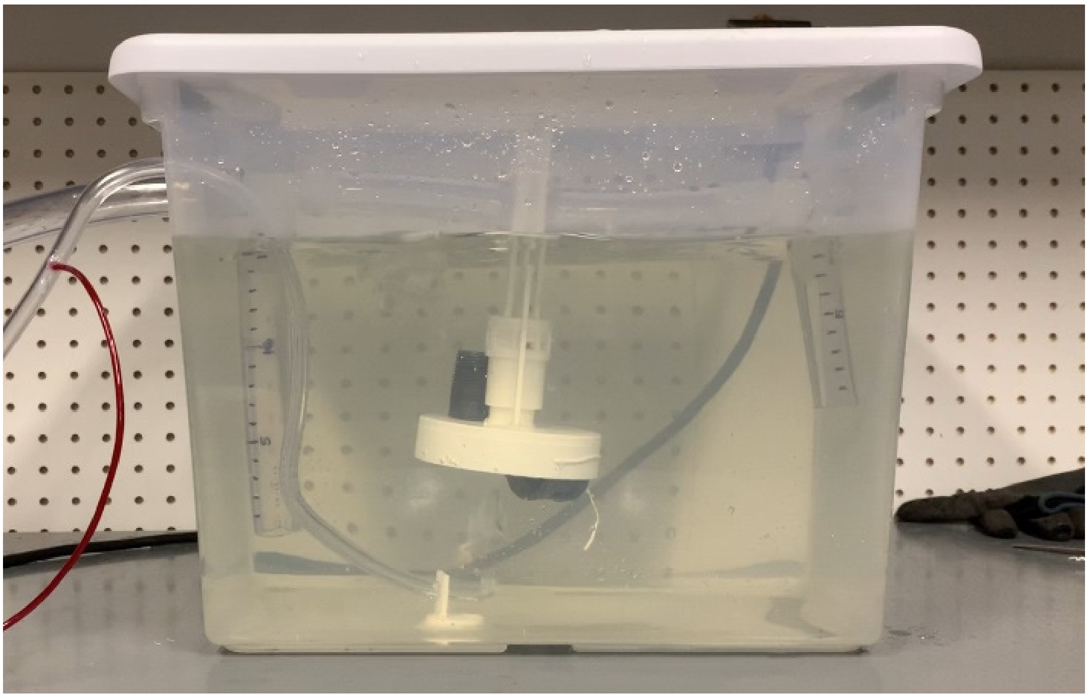

Figure S1. *The initial iteration of the BubbleVent, demonstrating the simplicity of the technology. The float mechanism driving the breathing circuit valve (BCV) is the white part in the middle of the tank, with the PIP and PEEP tubes located to the left and right, respectively. The gas flow into the float can be seen on the bottom of the tank. The entire ventilator can fit inside a commonly available polypropylene container.*

2.1. The Core Technology

The BubbleVent employs principles similar to those used in bubble continuous airway pressure (B-CPAP) apparatus, with an added mechanism to enable time-cycled high-pressure delivery that enables pressure-based ventilation. To deliver high-pressure breaths for intermittent mandatory ventilation (IMV), two airflows are utilized. One airflow serves as the breathing gas for the patient, and the second airflow drives the mechanism. The operational cycle of the mechanism is organized into four phases described below and illustrated in Figure S2.

- Phase 1 (P.1)—Air enters the float air chamber and displaces water in the cavity, increasing the buoyancy of the chamber. The bias flow passes out the PEEP tube due to its shallower depth;
- Phase 2 (P.2)—After a certain volume of gas is collected in the cavity, the float air chamber rises, closing the breathing circuit valve (BCV). Breathing gas still flows out the PEEP tube until the float no longer rises;
- Phase 3 (P.3)—The float air chamber reaches its upper limit and cannot rise further. The BCV closes and redirects the breathing gas from the PEEP tube to the PIP tube. Upon closing, the float valve button is actuated, and gas from the float air chamber begins to exit the via the float valve orifice outlet;

- Phase 4 (P.4)—Gas exits the float air chamber via the float valve orifice outlet, and the float air chamber refills with water, losing its buoyancy. The loss of buoyancy causes the float air chamber to sink, opening the BCV, and breathing gas begins flowing out of the PEEP tube again.

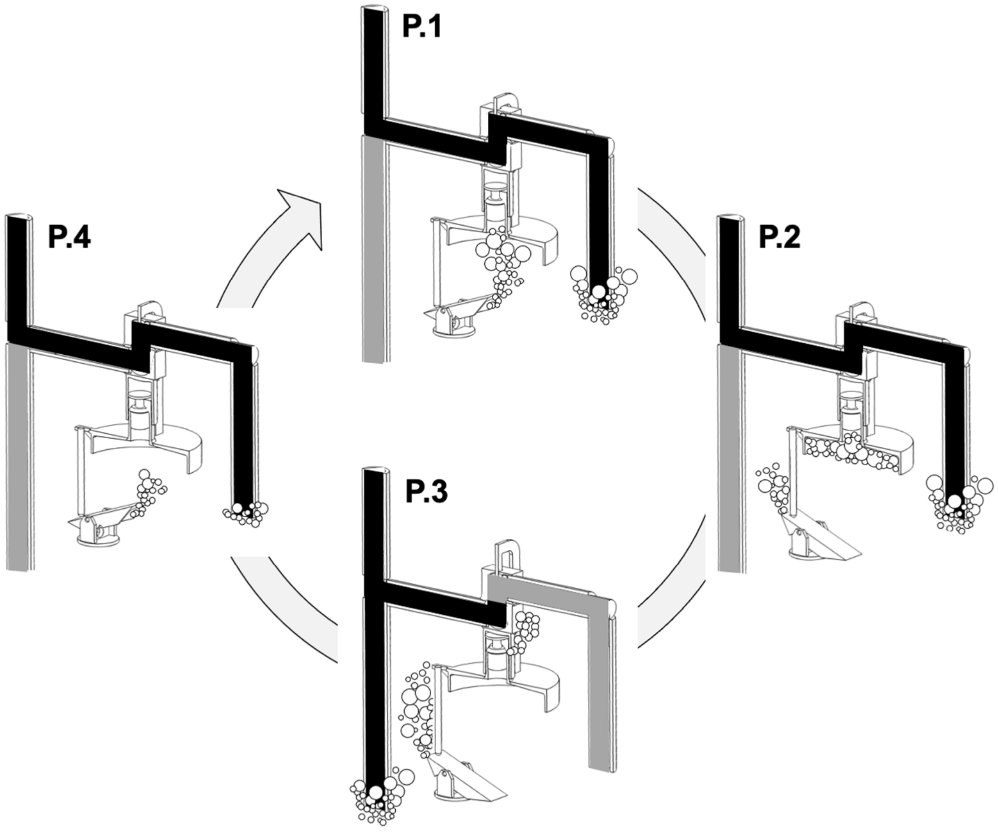

Figure S2. *Illustration of the essential mechanism of BCV, and four phases (a cycle) of BubbleVent operation. P.1: Float fills, the BCV is open, and breathing gas exits the PEEP tube. P.2: Float rises, BCV starts to close, and breathing gas continues to exit the PEEP tube. P.3: Float empties, and BCV is closed, breathing gas exits the PIP tube. P.4: Float falls, the BCV starts to open, breathing gas begins to exit PEEP tube.*

PEEP is provided when the breathing circuit valve (BCV) is open (P.1, P.2., and P.4), whereas PIP is provided when the BCV is closed (P.3). The core technology was based on the cycle of the float air chamber filling and emptying gas, which serves to open and close the BCV, redirecting gas intermittently between the PIP and PEEP tubes. Redirecting gas between PEEP and PIP pressures is the basis of positive pressure ventilation (PPV) therapy.

However, the mechanism described does not offer a way to control the time the BCV remains opened and closed. Without this independent control, the open-to-close ratio is constrained to approximately 1:1. This constraint prevents ventilation requirements being tailored to the patient, and increases the risk for barotrauma and volutrauma—an issue particularly important in the context of continuous mandatory ventilation (CMV). The key discovery of the authors is how to deliver effective ventilatory support using only hydro-pneumatic principles by providing reliable, independent control over inspiratory (T_I) and expiratory (T_E) timing.

2.2. Refining the Core Technology to Control Breathing Rates

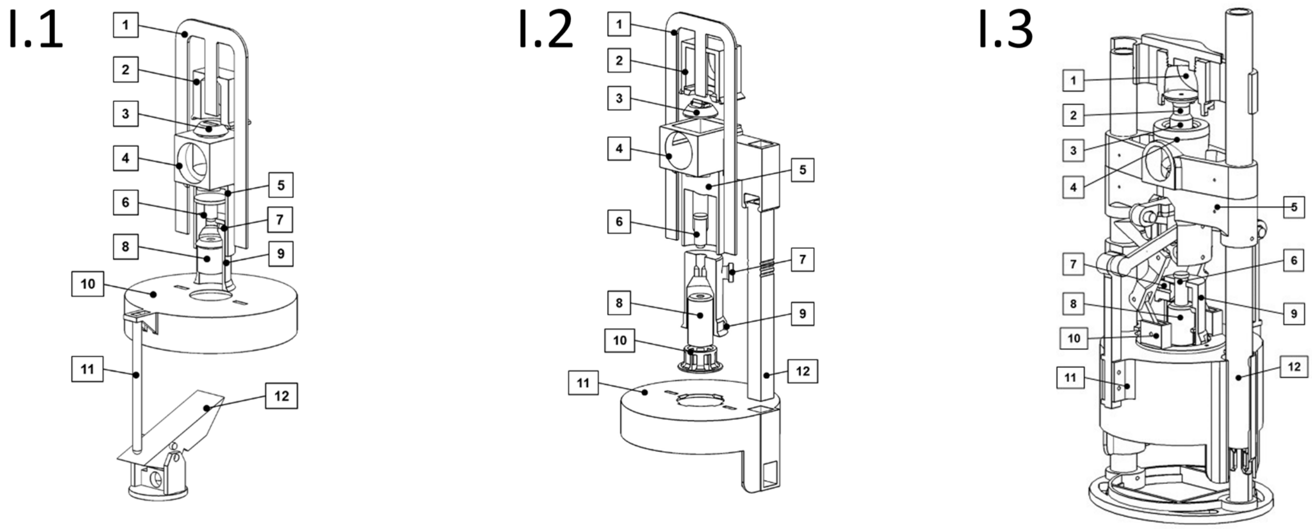

I.1

1. Breathing Circuit Valve (BCV) Control Rod
2. BCV Gas Outlet
3. BCV Valve Head
4. BCV Gas Inlet and Valve Seat
5. Float Valve Guide
6. Float Valve Button
7. Float Valve Outlet Orifice (fixed)
8. Float Valve Air Cylinder
9. Float Valve Housing
10. Float Air Chamber
11. Float Gas Flow Director Trigger
12. Float Gas Flow Director

I.2

1. Breathing Circuit Valve (BCV) Control Rod
2. BCV Gas Outlet
3. BCV Valve Head
4. BCV Gas Inlet and Valve Seat
5. Float Valve Guide
6. Float Valve Button
7. Float Valve Outlet Orifice (modular)
8. Float Valve Air Cylinder
9. Float Valve Housing
10. Float Valve Guide Ring
11. Float Air Chamber
12. Float Gas Flow Tube

I.3

1. BCV Gas Inlet
2. BCV Valve Head
3. Breathing Circuit Valve (BCV) Linkage Rod
4. BCV Gas Outlet and Valve Seat
5. BCV Linkage Guide
6. Float Valve Button
7. Float Valve Outlet Orifice (modular)
8. Float Valve Air Cylinder
9. Float Valve Housing
10. Timing Control Linkage
11. Float Air Chamber
12. Float Gas Flow Tube

Figure S3. *Illustrations of I.1, I.2, and I.3 BubbleVent Valve Systems. I.1 Focused on the timing of gas delivery to the float using the Float Gas Flow Director. I.2: Focused on controlling the rate of gas leaving the float using the Float Valve Outlet Orifice. I.3: Focused on controlling the volume of gas contained by the float timing control linkage.*

To reliably achieve this independent timing control, device development resulted into three key architectural iterations shown in Figure S3. Each iteration added design complexity, although these iterations also shared simple, common principles that harnessed fluid principles to deliver mechanical motion. In their simplest forms, all iterations use the buoyancy of an object to cause a valve to open and close (Figure S2), and use the rate at which buoyancy is achieved or lost to control the timing of the valve being opened and closed. This principle is also described in **Figure 1** of the primary text, and illustrated here in its earliest and most simple form.

The consistency and simplicity of the architecture of each iteration resulted in rapid improvements in device performance across the following areas:

1. The operational capability of the device, including the minimum and maximum delivered PIP and PEEP pressures, and the rate and independence of PIP and PEEP timing;
2. The reliability of the device including the consistency of delivered PIP and PEEP pressures, and consistency of PIP and PEEP timing;

3. Device ergonomics, including the process of adjusting the control variables such as delivered pressures, gas flow rates, and PIP and PEEP timing.

Unless otherwise stated, bias flow breathing gas used to test the I.1, I.2, and I.3 equipment was delivered at 50 LPM (pressure source regulated between 15 and 20 PSI). Gas delivered to the float air chamber ranged from 1 to 2 LPM. Performance of I.1, I.2, and I.3 during development was assessed by measuring pressure changes in the breathing gas with a lung bladder or a test lung connected to the system. System pressure was measured using custom low-pressure sensors (RM Aviation, New Carlisle OH). Data were digitized using a Mini-Digi 1B and collected in Axoscope 10 (Axon Instruments, Sunnydale CA) at 0.1 to 1.0 kHz.

We evaluated the timing of the apparatus as T_I as well as the cycle time. T_I was the time the float air chamber took to rise, completely close the BCV, and deliver PIP (Figure S2, P.3). The cycle time was the duration between equivalent phases in series, i.e., the duration between the start of P.1 and the time the next P.1 started. The cycle time included the total PIP and PEEP time, and the time taken to switch between these states.

2.2.1. Iteration 1 (I.1)

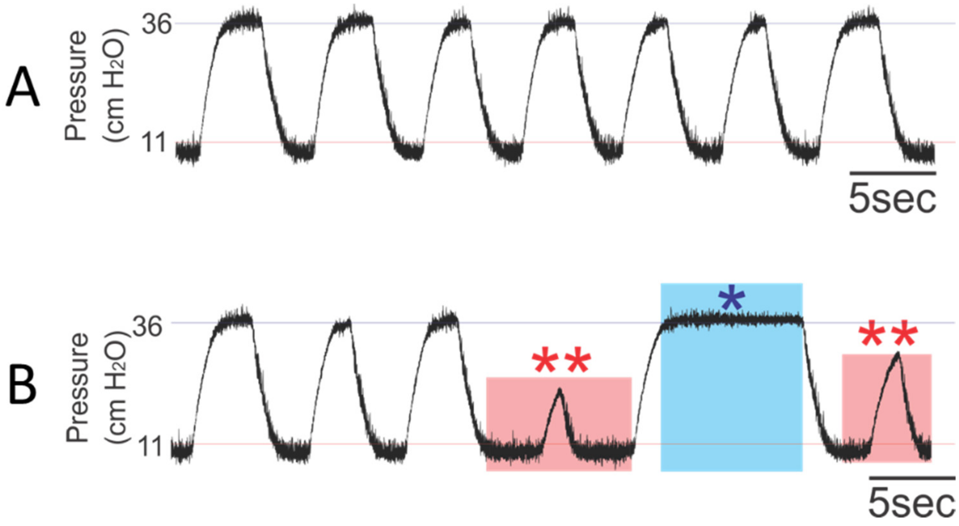

Figure S4. *Airway pressure tracings of I.1 illustrating normal operation (**A**) and a failure state (**B**). This is an indication of the incomplete or prolonged PIP periods identified when testing I.1*

The initial design of the BubbleVent employed mechanical control of the gas flow into the float air chamber. By determining when gas could enter the float air chamber, the float gas flow director (Figure , I.1, #12) helped to regulate T_I by determining when gas could enter the float air chamber. A fixed float valve outlet orifice allowed gas to leave the float air chamber at a consistent rate. PIP and PEEP timing was mostly reliable (Figure), but occasional failures from interactions between the components caused either incomplete PIP (Figure S4, dual asterisk) or unintended, prolonged periods of PIP (Figure S4, double asterisk). These prolonged periods of PIP often required manual adjustment of the flow director to restore normal function. The mechanical failures of I.1 caused unacceptable risk to the patient, and a new iteration was needed.

2.2.2. Iteration 2 (I.2)

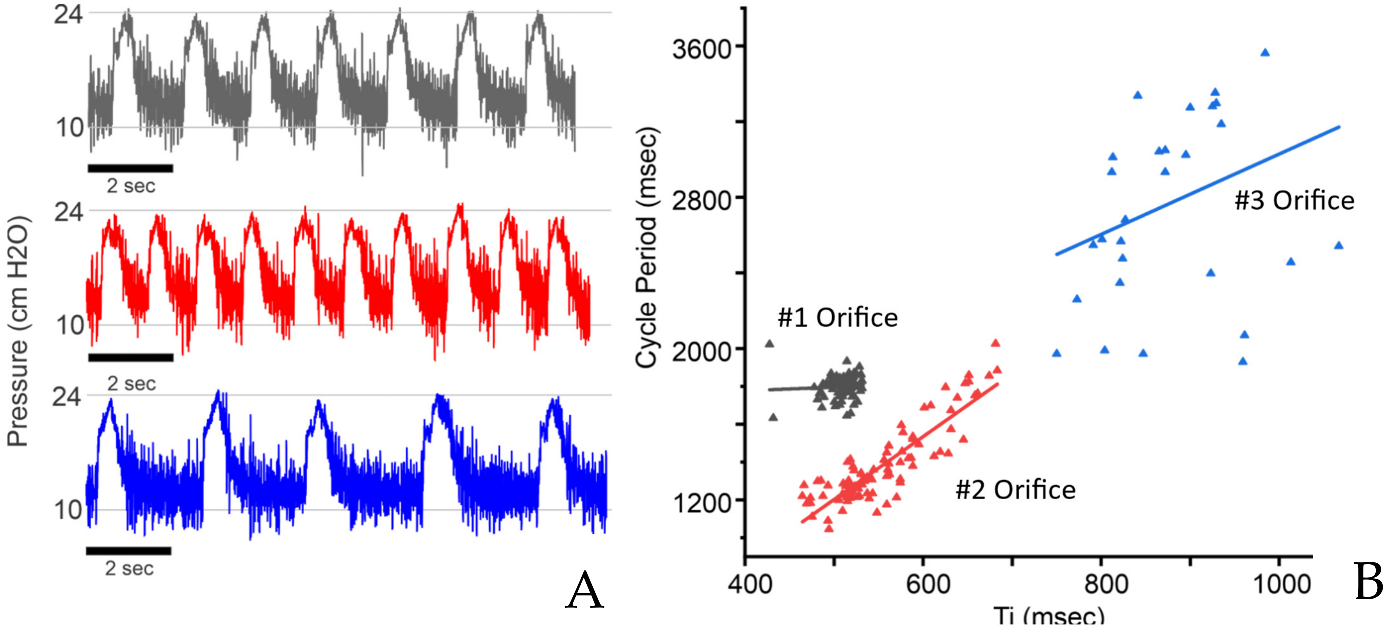

Figure S5. *(**A**): Airway pressure tracings of I.2 with three different float valve orifices. (**B**): The impact of changing the float valve orifice on T_I.*

To reduce the probability of mechanical failure and improve the timing control, I.2 integrated a float gas flow tube into the body of the valve system (Figure S3, I.2, #12) and added a modular float valve outlet orifice (Figure , I.2, #7). Integration of the float gas flow tube into the body of the valve system almost completely eliminated the failures observed with I.1. Moreover, the inclusion of the modular float valve outlet orifice enabled improved control of the T_I duration (Figure S5A). However, when using different float valve outlet orifice diameters at a constant gas flow rate into the float air chamber, both the T_I and T_E were changed (Figure S5B), indicating that the two periods were not being independently controlled.

2.2.4. Iteration 3 (I.3)

To enable independent control over T_I and cycle time, I.3 retained the modular float valve outlet orifice of I.2 and added a timing control linkage (Figure S3, I.3, #10). When in the locked position, the timing control linkage prevented the float air chamber from rising until gas had filled the float air chamber with a known volume. Once full, excess gas exited the float air chamber, unlocking the timing control linkage and allowing the buoyant float air chamber to rise and close the BCV.

By requiring a known volume of gas to be delivered to the float air chamber before it rose, changes to the flow rate of gas supplied to the float air chamber could precisely regulate the start of T_I. This allowed low gas flow rates to slowly fill the float air chamber, delaying the start of T_I; high flow rates cause the float to fill rapidly, hastening the start

of T_I. The float valve outlet orifice then functioned as in I.2, controlling the resistance to gas leaving the float and influencing the duration of T_I by controlling the time it took for the float air chamber to empty, the float to start to sink, and the BCV to open.

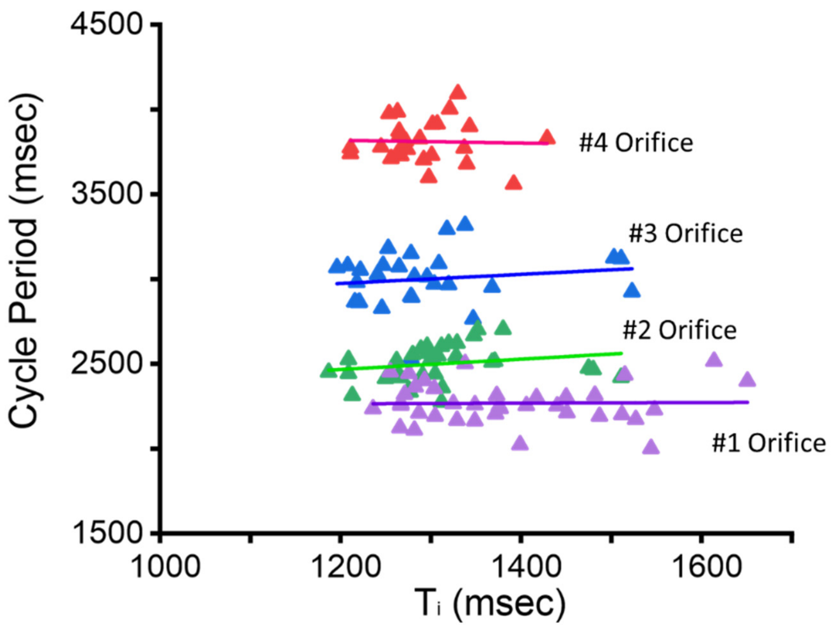

Figure S6. *Controlling the respiratory rate with I.3, illustrating the impact of the timing control linkage, the modular orifices, and the control of the float gas flow rate on the control of* T_I *and cycle time.*

Implemented the key elements of the three architecture iterations together established effective control of T_I and cycle time, i.e., regulating when and at what rate gas could enter the float air chamber, regulating when and at what rate gas could exit the float air chamber, and ensuring a consistent volume of gas was delivered to the float air chamber before it rose. The resulting architecture then allowed a range of inspiratory and expiratory timing ratios to be provided (Figure). I.3 resulted in a device architecture that formed the basis for the BubbleVent apparatus used in the testing described in the primary text for this supplement.

2.3. *Completing the Final BubbleVent Prototype*

2.3.1. Iteration 4 (I.4)

I.4 continued to undergo development from I.3 to refine the control of the T_I and T_E. The resulting I.4, known as the BubbleVent, also included a PIP timing control linkage to ensure a consistent volume of gas was left in the float air chamber before it sank. A supplemental mechanical mechanism—described as a "Float within Float"—was also added to trigger these linkages with clockwork reliability, based on the water level within the float air chamber.

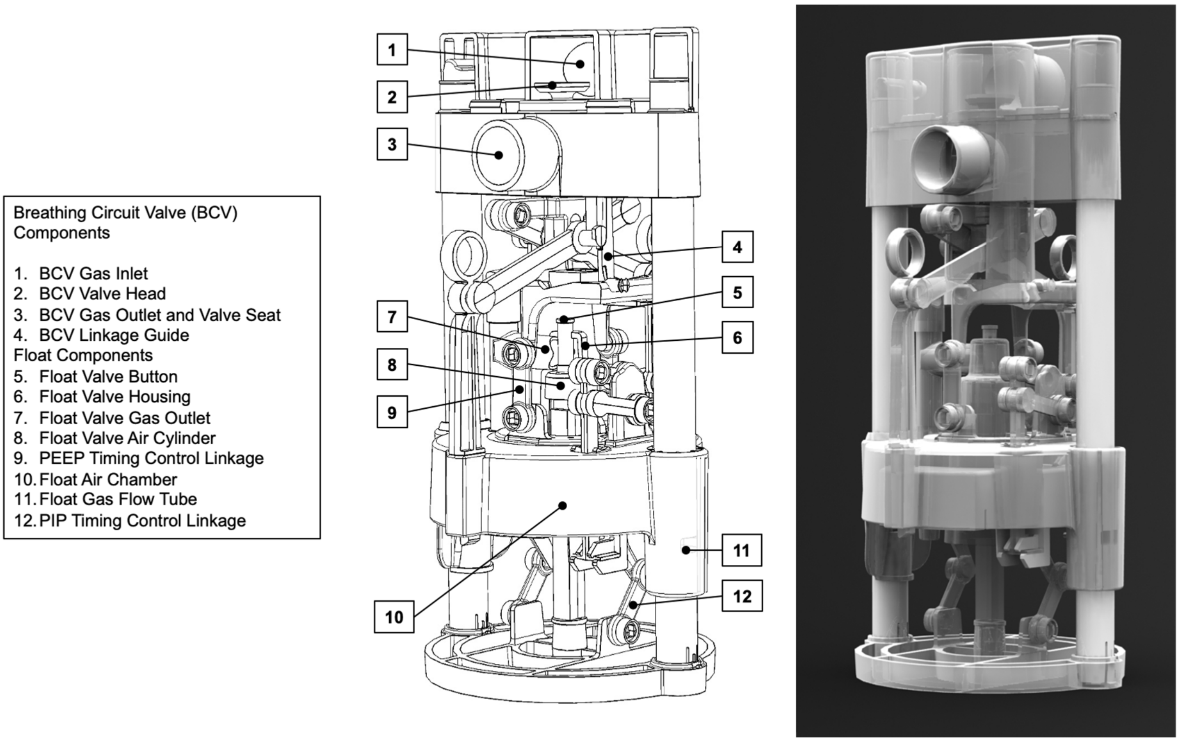

Figure S7. *Illustrations of the BubbleVent valve system for I.4. A PIP timing control linkage was added to the valve system to ensure a consistent volume of air was removed from the float valve chamber before the float could sink. The PEEP and PIP timing control linkages shared a common design and parts.*

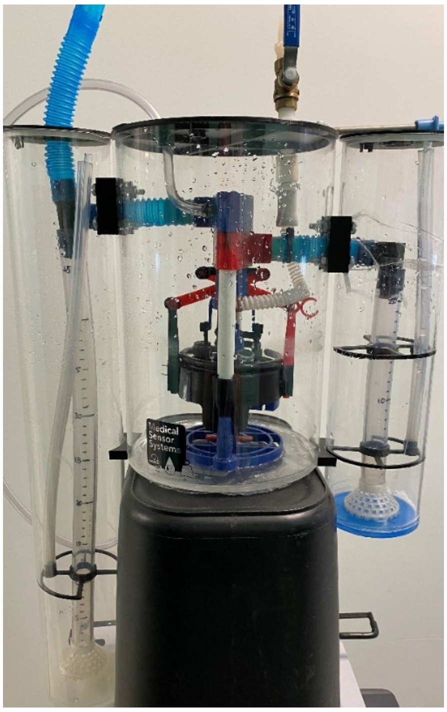

Figure S8. *Picture of the BubbleVent prototype used for the study. The three water tanks enabled individual control of PIP, PEEP, T_I, and T_E.*

2.3.2. Addition of Sequoia

Data on the performance of the BubbleVent were collected using Sequoia (Medical Sensor Systems, Seattle, WA, USA). Sequoia is a low-cost sensor array platform designed to monitor physical variables in the healthcare setting. Sequoia is capable of sampling, synchronizing, processing, and storing up to six different real-time signals sampled at 100 Hz. Sequoia was adapted to support airway pressure (P_{AW}), bias flow, RR, and T_I monitoring of the BubbleVent system. This adaptation of Sequoia was termed the BubbleVent Monitoring System (BVMS). The data were cleaned and restructured before being transferred to a personal computer via USB. The sampling rate of the system was 100 Hz. Real-time BVMS data were compared with real-time data generated by the ASL 5000 Test Lung (Ingmar Medical, Pittsburgh, Pennsylvania).

Hardware: Airway pressure, bias flow, T_I, and RR were monitored using three sensors. Paw was monitored using a Honeywell TurStability® board-mounted pressure sensor (SSCDRRN010ND2A3, Honeywell, Golden Valley, MN, USA). Bias flow was measured with Sensirion Mass Flow Sensor (SFM3000, Sensirion AG, Switzerland).

T_I and RR were determined using an optical switch (Lerdge Optical Endstop 4001, R REIFENG, China). The switch was fixed onto the Valve System Tank Lid. A shaft was secured to the float air chamber and protruded through the lid, passing near the optical switch. The switch, therefore, indirectly monitored the BVC opening and closing, and was used as a surrogate for breath time. Actuation of the switch occurred when the float was up, closing the BVC and initiating inhalation. When the float emptied and sunk to its low position, the BVC opened and initiated exhalation. The time of inhalation and exhalation was recorded, and RR was calculated from those time estimates.

All sensors were communicated via the I2C protocol and aggregated through a multiplexor (TCA9548A, Texas Instruments, Dallas, USA) before being passed along to the microcontroller unit (Arduino Micro, Arduino, Italy). The data were cleaned and restructured before being transferred to a personal computer via USB. The sampling rate of the system was 100 Hz.

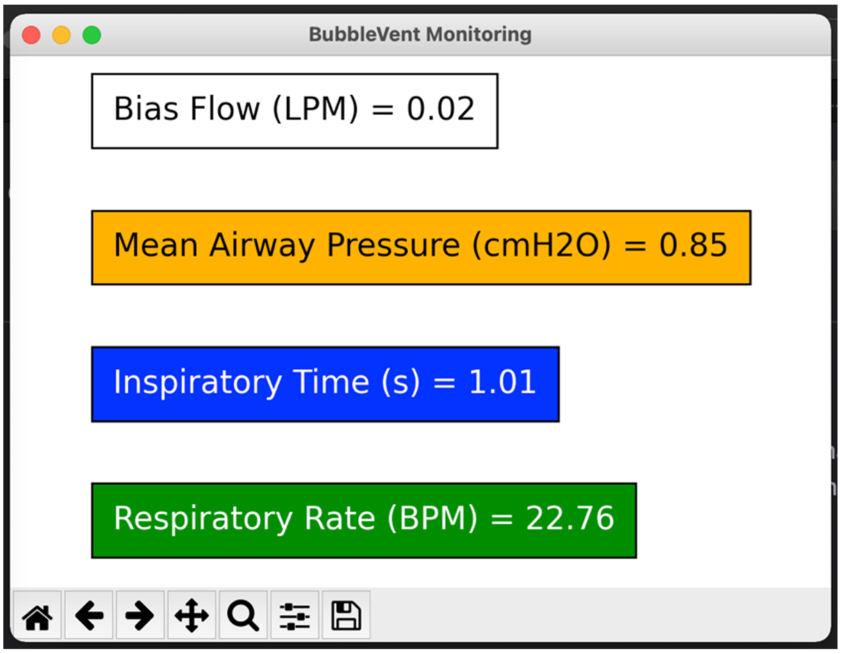

Figure S9. *Interface of the BubbleVent Monitoring System (BVMS). Any adjustments made to the BubbleVent are reflected in the window.*

Software: All software was developed using Python, leveraging the Numpy, Pandas, PySerial, Matplotlib, and Tkinter libraries. The data streaming from the serial port were parsed and stored as a CSV file. The Paw and Bias Flow signals were recorded and displayed in real time. An alarm was triggered if Paw fell below 0cmH_2O or increased to greater than 60cmH_2O. RR and T_I were computed from signals generated by the optical switch and were displayed on the top of the plot. For this test, the software was executed and data were stored on a MacBook Pro 2014 with 16GB RAM on an Intel i7 processor. Data were displayed on a display as shown in Figure S9.

3. ISO 80601 Pressure Ventilation Testing

3.1. Methods

To test the performance of the BubbleVent against a critical care ventilator and to determine whether it met safety and efficacy specifications, a ventilator test outlined by the International Standards Organization (ISO) was conducted. Following the pressure-control ventilator testing protocols outlined in ISO Standard 80601, the BubbleVent and the Draeger were tested using the ASL 5000 set to various resistances and compliances (Table S1). T_I and RR were maintained constant at 1.0 s and 20 BPM, respectively, throughout testing. The Draeger VN500 was set to PC-AC mode without a volume guarantee in order to operate in a similar fashion to the BubbleVent. For each PIP and PEEP setting, the BubbleVent water level in the PIP and PEEP tanks were titrated to the appropriate water level, which ensured the closest possible output to the intended set pressure. Bias flow was kept constant at 50 LPM for all eight tests, and was selected because this was the lowest flow that ensured bubbling throughout the PIP and PEEP tubes at every pressure level.

Table S1. Lung model parameters and ventilator settings for the first eight tests of the ISO 80601 pressure support ventilation.

Test #	Lung Volume (mL)	Lung Model Compliance (mL/hPa)	Lung Model Resistance (hPa/L/s)	RR (BPM)	T_I (s)	PIP (cmH_2O)	PEEP (cmH_2O)
1	500	50	5	20	1.0	15	5
2	500	50	20	20	1.0	25	10
3	500	20	5	20	1.0	30	5
4	500	20	20	20	1.0	35	10
5	300	20	20	20	1.0	20	5
6	300	20	50	20	1.0	35	10
7	300	10	50	20	1.0	35	5
8	200	10	20	20	1.0	35	10

PEEP, positive end expiratory pressure; PIP, peak inspiratory pressure; T_I, inspiratory time; RR, respiratory rate.

3.2. Results of ISO 80601 Benchmark Testing

Tables S2, S3, and S4 below show the outcomes of the ISO 80601 testing. Table S2 focuses on the timing of the breaths and consistency over the measured epoch. Table S3 focuses on the pressure delivery. Table S4 focuses on volume delivery. The percentage error in the tables was the error relative to the intended values in Table S1.

Table S2. Timing outcomes of the ISO 80601 test. Measured T_I and RR for the patient at different settings of compliance and resistance. T_I and RR were set using the BubbleVent Monitoring System (BVMS) and measured using the ASL 5000. Values are the mean ± SD.

Test #	BV T_I (s)	BV T_I % Error	VN500 T_I (s)	VN500 T_I % Error	BV RR (BPM)	BV RR % Error	VN500 RR (BPM)	VN500 RR % Error
1	0.96 ± 0.021	-3.76	0.99 ± 0.002	-0.75	19.86 ± 0.061	-0.72	20.00 ± 0.003	-0.01
2	1.03 ± 0.054	3.23	0.98 ± 0.002	-2.30	20.68 ± 0.075	3.38	20.00 ± 0.002	0.00
3	0.95 ± 0.054	-4.69	0.99 ± 0.003	-0.64	19.83 ± 0.078	-0.87	20.00 ± 0.003	0.00
4	1.02 ± 0.046	2.13	1.00 ± 0.001	0.03	20.08 ± 0.060	0.42	20.00 ± 0.002	0.00
5	0.98 ± 0.044	-2.53	1.00 ± 0.002	-0.15	20.76 ± 0.055	3.79	20.00 ± 0.002	-0.01
6	1.05 ± 0.050	5.42	0.99 ± 0.001	-0.99	19.88 ± 0.074	-0.60	20.00 ± 0.001	-0.01
7	1.04 ± 0.041	4.13	1.00 ± 0.001	-0.22	20.25 ± 0.051	1.23	20.00 ± 0.001	0.00
8	1.00 ± 0.040	0.42	0.99 ± 0.002	-0.27	20.62 ± 0.057	3.11	20.00 ± 0.003	-0.01

BV, BubbleVent; T_I, inspiratory time; RR, respiratory rate.

Table S3. Pressure outcomes of ISO 80601 test. Measured PIP and PEEP for the patient at different settings of compliance and resistance. PIP and PEEP were titrated using the BubbleVent Monitoring System (BVMS) and measured using the ASL 5000. Values are the mean ± SD.

Test #	BV PIP (cmH_2O)	BV PIP % Error	VN500 PIP (cmH_2O)	VN500 PIP % Error	BV PEEP (cmH_2O)	BV PEEP % Error	VN500 PEEP (cmH_2O)	VN500 PEEP % Error
1	15.06 ± 0.248	0.43	14.73 ± 0.010	-1.78	4.89 ± 0.134	-2.06	5.19 ± 0.010	3.82
2	24.49 ± 0.157	-2.01	24.51 ± 0.017	-1.97	9.90 ± 0.714	-0.96	10.85 ± 0.025	8.58
3	29.72 ± 0.292	-0.93	30.44 ± 0.013	1.48	5.02 ± 0.177	0.52	5.34 ± 0.012	6.83
4	34.65 ± 0.413	-1.00	34.97 ± 0.013	-0.08	10.15 ± 0.436	1.56	10.26 ± 0.011	2.66
5	20.54 ± 0.635	2.68	19.92 ± 0.010	-0.37	4.76 ± 0.260	-4.68	5.12 ± 0.008	2.42
6	35.47 ± 0.253	1.36	35.14 ± 0.039	0.40	10.01 ± 0.783	0.07	10.55 ± 0.02	5.57
7	34.58 ± 0.643	-1.17	35.06 ± 0.052	0.18	4.95 ± 0.284	-1.05	5.19 ± 0.012	3.95
8	35.01 ± 0.573	0.04	35.44 ± 0.010	1.26	9.65 ± 0.457	-3.43	10.26 ± 0.011	2.69

BV, BubbleVent; PIP, peak inspiratory pressure; PEEP, positive end expiratory pressure.

Table S4. Volume outcomes of ISO 80601 test. Measured volume at the patient using the ASL 5000 at different settings of compliance and resistance. Values are the mean ± SD.

**Test #**	**BV V_T (cmH_2O)**	**BV V_T % Error**	**VN500 V_T (cmH_2O)**	**VN500 V_T % Error**
1	435.69 ± 3.79	-12.86	441.15 ± 1.03	-11.77
2	305.01 ± 10.78	-39.00	360.48 ± 0.42	-27.90
3	508.81 ± 6.25	1.76	534.55 ± 0.26	6.91
4	435.11 ± 7.39	-12.98	454.37 ± 0.34	-9.13
5	243.03 ± 4.36	-18.99	269.06 ± 0.10	-10.31
6	220.64 ± 5.38	-26.45	277.82 ± 0.23	-7.39
7	257.45 ± 3.77	-14.18	267.91 ± 0.58	-10.69
8	260.15 ± 2.11	30.08	275.21 ± 0.08	37.61

BV, BubbleVent; V_T, tidal volume
